# Modulation of Drug Resistance by Furanochromones in *NorA* Overexpressing *Staphylococcus Aureus*

**DOI:** 10.1155/2022/9244500

**Published:** 2022-09-17

**Authors:** Damara F. Rodrigues, Nathalie H. P. B. Borges, Carlos Emídio S. Nogueira, Josean F. Tavares, Daniel Dias Rufino Arcanjo, Humberto M. Barreto, José P. Siqueira-Junior

**Affiliations:** ^1^Laboratory of Genetics of Microorganisms, Department of Molecular Biology, Federal University of Paraiba, João Pessoa, PB, Brazil; ^2^Department of Biological Chemistry, Regional University of Cariri, Crato, CE, Brazil; ^3^Department Pharmaceutical Science, Federal University of Paraíba, João Pessoa, PB, Brazil; ^4^Laboratory of Functional and Molecular Studies on Physiopharmacology (LAFMOL), Department of Biophysics and Physiology, Federal University of Piauí, Teresina, PI, Brazil; ^5^Laboratory of Research in Microbiology, Department of Parasitology and Microbiology, Federal University of Piaui, Teresina, PI, Brazil

## Abstract

Khellin and visnagin are natural furanochromones that photoreact with DNA. Khellin has been used in the treatment of vitiligo and psoriasis, as well as in the treatment of angina pectoris and asthma due to its potent action as a coronary vasodilator and antispasmodic agent. The present study aimed to investigate whether the compounds khellin and visnagin act as inhibitors of NorA protein, an efflux pump overproduced by the strain of *Staphylococcus aureus* SA-1199B that confers resistance to the fluoroquinolones, such as norfloxacin and ciprofloxacin. These substances alone did not show antibacterial activity against the strain tested. On the other hand, when these compounds were added to the culture medium at subinhibitory concentration, they were able to reduce the minimum inhibitory concentration (MIC) of norfloxacin, ethidium bromide, as well as berberine, suggesting that these compounds are modulating agents of norfloxacin resistance, possibly due to NorA inhibition. Molecular docking analysis showed that both khellin and visnagin form hydrogen bonds with Arg310, an important residue in the interaction between NorA and its substrates, supporting the hypothesis that these compounds are NorA inhibitors. These results suggest a possible application of khellin and visnagin as adjuvants to norfloxacin in the treatment of infections caused by strains of *S. aureus* that overproduce NorA.

## 1. Introduction

Infectious diseases caused by multidrug-resistant bacteria have become a global health problem, leading to high mortality rates [1], and high costs for healthy systems [2]. Bacterial resistance is an adaptive response caused by the intensive use of antibacterial agents in the most diverse areas, including in the human and veterinary medicines, as well as animal feed supplementation, selecting the most adapted strains [3]. Several mechanisms of antibiotic resistance have been demonstrated in bacteria, such as reducing the permeability of bacteria to antimicrobials, enzymatic modification of the antibiotic target, antibiotic modification, or degradation, as well as drug extrusion by efflux pumps [4].

Efflux pumps are transmembrane proteins that account for much of the bacterial resistance since they pump antimicrobial agents out of the cell [5]. Some of these pumps are specific for a given compound or class of compounds, whereas others remove a variety of structurally unrelated antimicrobial compounds [6, 7]. Several efflux pumps related to drug resistance in *Staphylococcus aureus* have already been identified, including MsrA, MepA, LmrS, MdeA, NorA, NorB, NorC, QacA, and QacC [8]. NorA is a proton-dependent multidrug efflux pump that belongs to Major Facilitator Superfamily (MFS) and it confers resistance to hydrophilic fluoroquinolones, such as norfloxacin and ciprofloxacin, as well as to biocide agents, such as ethidium bromide, acriflavine, and benzalkoniun chloride [9–11].

Resistance-modifying agents/modulators are compounds that potentiate the activity of an antibiotic against resistant strains, and some of these agents may act as efflux pump inhibitors (EPIs), as in the case of several naturally occurring compounds from plants [12–16]. Various phytocompounds have been studied for its ability to inhibit *S. aureus* efflux pumps, including terpenes such as eugenol [17], carvacrol, thymol [18], estragole [19], *α*-pinene, and limonene [20]. Inhibition of NorA has also been reported for ferulic acid and its esterified derivatives [21], chalcones [22], and vitamin K3 [23]. On the other hand, various synthetic EPIs have been reported as efflux pump inhibitors [24–26].

Chromone (1,4-benzopyrone) is a derivative of benzopyran with a substituted keto group on the pyran ring, being an isomer of coumarin (1,2 benzopyrone). Visnagin and khellin are two naturally occurring furanochromones able to photoreact with DNA [27]. Khellin and visnagin derivatives have anti-inflammatory and analgesic activity [28], epidermal growth factor inhibitory activity [29], as well as light mediated antimicrobial activity against *Escherichia coli* and *Fusarium culmorum* L. [30]. Khellin has been used in the treatment of vitiligo, and psoriasis [31], as well as in the treatment of angina pectoris and asthma due to its potent action as a coronary vasodilator and antispasmodic agent [32, 33]. Furthermore, khellin also has been used in the treatment of kidney stones [34].

Furochromones, as well as furocoumarins, are widely studied for their photoactive properties [27, 35]. The modulating activity of drug resistance by furochromones has not yet been reported, requiring the development of studies with these molecules as potential efflux pump inhibitors.

**Figure 1:**
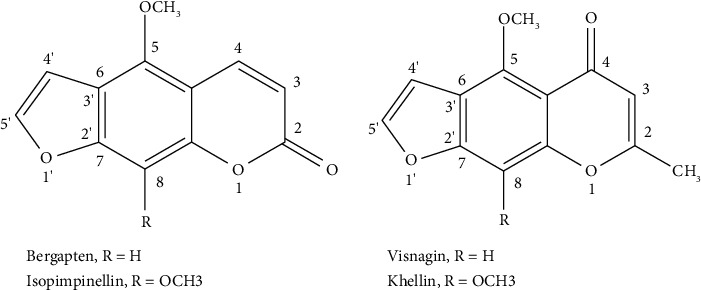
Chemical structure of furanocoumarins (bergapten and isopimpinellin) and furanochromones (visnagin and khellin).

In an ongoing project to evaluate coumarins as modulators of antibiotic resistance, the modulatory activity of semisynthetic and commercial coumarins [36], as well as of furanocoumarins isolated from Rutaceae species [37] has been demonstrated. Still regarding the furanocoumarins, bergapten, and isopimpinellin do not modulate the resistance to norfloxacin and to ethidium bromide in an effluxing *Staphylococcus aureus* strain. Although furanochromones visnagin and khellin present similar molecular structures to bergapten and isopimpinellin, respectively (Figure 1), these compounds were considered different enough from the furacoumarins to justify the evaluate these homologous furanochromones (visnagin and khellin) as modulators of antibiotic resistance using an effluxing *Staphylococcus aureus* strain.

## 2. Material and Methods

### 2.1. Chemicals

The stock solution of norfloxacin was prepared in a mixture of 1 M NaOH and sterile distilled water (1 : 9 proportion). The stock solution of ethidium bromide (EtBr) and berberine were prepared in distilled water. The stock solution of the furanochromones—khellin and visnagin, were prepared in DMSO which, at its highest final concentration after dilution in the broth (4%), displayed no inhibition of bacterial growth. Chlorpromazine was prepared in sterile distilled water. All drugs were from Sigma-Aldrich, USA.

### 2.2. Bacteria

The SA-1199B strain of *S. aureus* was used as it overexpresses the *norA* gene. This gene encodes the NorA efflux protein that extrudes not only norfloxacin but several compounds, such as: hydrophilic fluoroquinolones, quaternary ammonium compounds benzalkonium chloride and cetrimide, intercalating dyes acriflavine and ethidium bromide [10], as well as the alkaloid berberine [38]. The strain, provided by Professor Simon Gibbons (University College London, UK), was maintained in blood agar base (Laboratories Difco Ltda., Brazil) slants, and prior to use, the cells were grown overnight at 37°C in brain heart infusion broth (BHI–Difco Ltda., Brazil).

### 2.3. Drug Susceptibility Testing and Modulation Assay

The minimum inhibitory concentrations (MICs) of norfloxacin, pefloxacin, ethidium bromide, and furanochromones were determined in BHI by the microdilution assay using a suspension of ca. 10^5^ cfu/mL and a drug concentration range of 1024–1 *μ*g/mL (two-fold serial dilutions). The MIC was defined as the lowest concentration at which no growth is observed. The detection was performed after the addition of resazurin at 0.01%. For the evaluation of furanochromones as a modulator of drug resistance, the “modulation assay” was used, a method that has been widely applied to identify potential EPIs [12] i.e. the MICs of norfloxacin, pefloxacin, ethidium bromide, and berberine were determined in the presence of furanochromones at a subinhibitory concentration (MIC 1/4). Chlorpromazine, a known NorA inhibitor [39], was used as a positive control.

### 2.4. Docking Procedure

The NorA model for the docking procedure was created by retrieving the NorA sequence of *S. aureus* 1199 strain from the Universal Protein Resource database (Uniprot, Entry Q03325). Then, the SWISS-MODEL [40] service was used to build the homology model. Out of the templates generated, the one with the best GMQE (Global Model Quality Estimation) score was the one based on the structure of the *Escherichia coli* YajR transporter (PDB-ID: 3wdo). The Molprobity [41] service was used for the protonation of the NorA model. For the docking procedure, which was carried out using the Autodock Vina [42] software, the grid box was defined as a 20Åx20Åx20Å box around the geometrical center of the model. Partial Gasteiger charges were added to ligand atoms, nonpolar hydrogen atoms were mixed while all other parameters were kept at their default values. Best results were chosen based on the binding score.

## 3. Results and Discussion

According to a previous study, the antimicrobial activity of an isolated compound must be considered significant if its MIC value is ≤10 *μ*g/mL [43]. Both furanochromones khellin, and visnagin showed MIC values ≥1024 *μ*g/mL. These results indicate that the tested compounds were inactive against the *S. aureus* strain used.

Despite not showing activity against this specific strain, addition of these compounds to the growth medium at 256 *μ*g/mL (MIC 1/4) potentiated the antibacterial effect of norfloxacin against the SA-1199B strain, reducing the MIC values for norfloxacin by at least two-fold (Table 1). Modulating effect on the resistance to norfloxacin could be explained by inhibition of NorA efflux pump overproduced by SA-1199B, as already reported for several compound classes, such as alkamides [18], chalcones [44–46], flavonoids [47–50], and lignans [51]. In fact, visnagin, and khellin showed a modulating effect like that exhibited by the known NorA inhibitor chlorpromazine.

To investigate a potential action as NorA inhibitors, assays were performed replacing norfloxacin by two known NorA substrates: EtBr [52] and berberine [53]. EtBr is a well-known substrate for the NorA efflux protein, and active efflux is the only known mechanism of resistance to this DNA-intercalating dye [54]. Therefore, the use of EtBr against SA-1199B is enough to demonstrate that the compounds evaluated here modulated the resistance to norfloxacin by efflux pump inhibition. Results showed that compounds tested also reduced MIC values for EtBr and berberine by at least two-fold. It is worth noting the results obtained with visnagin regarding EtBr (Table 1). All experiments were carried out at least twice with consistent results suggesting that compounds tested could be NorA inhibitors.

**Figure 2:**
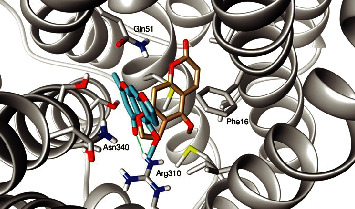
The best poses of visnagin (blue) and bergapten (golden) on the binding site of the NorA model. Hydrogen bond with Arg310 depicted in green.

**Figure 3:**
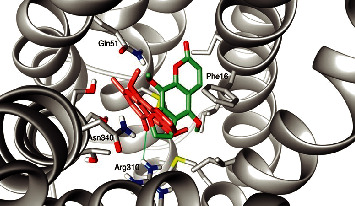
The best poses of khellin (orange) and isopimpinellin (green) on the binding site of the NorA model. Hydrogen bond with Arg310 depicted in green.

A previous study verified that furanocoumarins bergapten and isopimpinellin did not modulate the antibacterial activity of norfloxacin against the strain evaluated [37]. To understand the differences in efflux pump inhibition (EPI) capabilities of furanocoumarins and furanochromones, a molecular docking study against the NorA efflux pump model was conducted. A comparison of the binding pose of visnagin vs bergapten and khellin vs isopimpinellin is shown in Figures 2 and 3, respectively.

**Figure 4:**
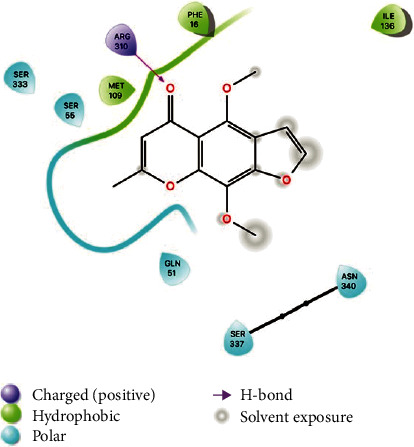
Protein-ligand interaction diagram of khellin on the binding site of the NorA model.

Interestingly, both furanochromones dock in almost the same fashion and interact with the same residues. In particular, both interact through hydrogen bonds with Arg310. There are close contacts with Phe16, Asn340, and Gln51. The binding pocket of NorA is described as being composed by Ile19, Ile23, Gln51, Met109, Ile136, Thr211, Arg310, Ile313, Thr314, Asn332, Ser333, Ser337, Asn340, and Phe341, among others. The furanochromones make close contact with most of these, as can be seen in the protein-ligand interaction diagram in Figure 4. Not only that but a previous study described a chalcone with NorA inhibition properties that interacts with Arg310 through a hydrogen bond [55]. There are also imidazolines EPIs that bind to this same region of the binding site and interact with Arg310 through hydrogen bonds [19]. Interaction through H-bond with Arg310 also was reported to aminoguanidine hydrazones [7, 56]. These results suggest that Arg310 is an important amino acid residue for NorA substrate recognition.

**Figure 5:**
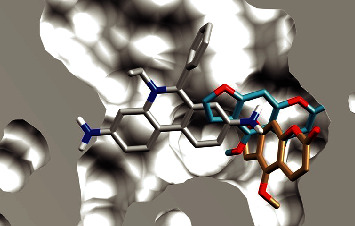
Binding poses of EtBr (white), visnagin (blue), and bergapten (golden) on the binding site of the NorA model.

On the other hand, furanocoumarins do not interact with Arg310. Also, the Arg310 hydrogen bond anchors the furanochromones in a way that hinders the binding of EtBr, as can be seen in Figure 5. As such, the binding pose of furanocoumarins shows almost no overlap with the position of EtBr. It could, thus, be argued that the furanochromones could act as efflux pump inhibitors through competition in contrast with furanocoumarins, supporting the experimental results shown before.

**Figure 6:**
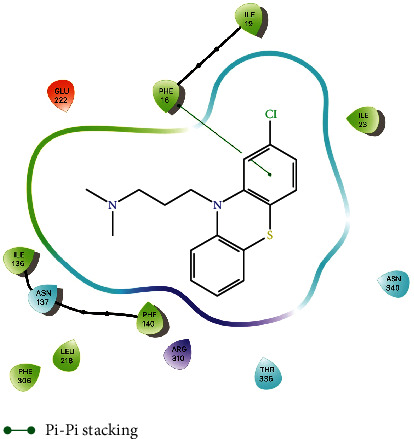
Protein-ligand interaction diagram of chlorpromazine on the binding site of the NorA model.

We also compared the docked pose of other known efflux pump inhibitors, such as chlorpromazine (Figure 6), as well as piperine (Figure S1), verapamil (Figure S2), and reserpine (Figure S3). Chlorpromazine binds to the same region of the binding site, interacting not only with Arg310, but with Phe16, Asn340, Ile136 and others. More importantly, its best pose overlaps with that of EtBr in a similar fashion as those of furanochromones.

Also, other EPIs such as piperine and verapamil also bind to the same region of the binding site (Figures S1 and S2), as both interact with Arg310, Phe16, Gln51, etc. The binding pose of reserpine, on the other hand, is a bit different. As it is much larger than the previously mentioned EPIs, its interaction site goes from Ile136, Asn340, and Phe16 to other parts of the binding site, such as Ala48 and Met52, its sheer size contributing to inhibition as much as its interactions (Figure S3).

Our results indicate that khellin and visnagin could be applied in combination with norfloxacin against NorA overproducer *S. aureus* strains. Khellin showed a low-level toxicity in humans, at an average daily dose of 120 mg, administered orally [57]. Liver and dermal histological and pathological analyses demonstrated that hydroxyethyl cellulose hydrogels based on khellin loaded in the ASC10 ascosomes have no toxic effects in rats [58]. On the other hand, both khellin and visnagin showed a strong cytotoxic activity (IC_50_ ranging between 12.54 and 17.53 *µ*g/mL) on breast cancer (MCF-7) and hepatocellular carcinoma (Hep G2) cell lines [59]. Therefore, studies *in vivo* will be necessary to evaluate the safety of using khellin or visnagin combined with norfloxacin.

## 4. Conclusion

The furanochromones Khellin and Visnagin did not show any antibacterial activity against *S. aureus* resistant to norfloxacin by efflux pump mechanism. However, both compounds reduced the MIC of norfloxacin at subinhibitory concentration. Furthermore, khellin and visnagin modulated the resistance to EtBr and berberine, suggesting a possible inhibition of NorA efflux pump overproduced by *S. aureus* SA-1199B strain. Molecular docking analysis showed that both khellin and visnagin form hydrogen bonds with Arg310, an important residue in the interaction between NorA and its substrates, supporting the hypothesis that these compounds could be NorA inhibitors. Results obtained at this work are quite promising, which may stimulate future studies about the use of natural products concerning the viability of its use against microbial resistance.

## Figures and Tables

**Table 1:** Minimum inhibitory concentrations (*μ*g/mL) of norfloxacin, ethidium bromide, berberine, and pefloxacin against *Staphylococcus aureus* strain SA-1199B in the absence or presence of khellin, visnagin, and chlorpromazine.

Drugs	Alone	+Khellin	+Visnagin	+Chlorpromazine
Norfloxacin	64	32 (2)*∗*	32 (2)	16 (4)
Ethidium bromide	64	32 (2)	16 (4)	8 (8)
Berberine	512	256 (2)	256 (2)	512
Pefloxacin	16	16	16	16

*∗*(fold reduction in MIC).

## Data Availability

The data used to support the findings of this study are available from the corresponding author upon request .

## Supplementary Materials

Supplementary Materials Figure S1: protein-ligand interaction diagram of piperine on the binding site of the NorA model. Figure S2: protein-ligand interaction diagram of verapamil on the binding site of the NorA model. Figure S3: protein-ligand interaction diagram of reserpine on the binding site of the NorA model.

## References

[1] World Health Organization (2014). Antimicrobial resistance: global report on surveillance. https://www.who.int/publications/i/item/9789241564748.

[2] O’Neill J. (2014). Antimicrobial resistance: tackling a crisis for the health and wealth of nations. https://amr-review.org.

[3] Venter H., Henningsen M. L., Begg S. L. (2017). Antimicrobial resistance in healthcare, agriculture and the environment: the biochemistry behind the headlines. *Essays in Biochemistry*.

[4] Kakoullis L., Papachristodoulou E., Chra P., Panos G. (2021). Mechanisms of antibiotic resistance in important Gram-positive and Gram-negative pathogens and novel antibiotic solutions. *Antibiotics*.

[5] Du D., Wang-Kan X., Neuberger A. (2018). Multidrug efflux pumps: structure, function and regulation. *Nature Reviews Microbiology*.

[6] Spengler G., Kincses A., Gajdács M., Amaral L. (2017). New roads leading to old destinations: efflux pumps as targets to reverse multidrug resistance in bacteria. *Molecules*.

[7] Pathania R., Sharma A., Gupta V. (2019). Efflux pump inhibitors for bacterial pathogens: from bench to bedside. *Indian Journal of Medical Research*.

[8] Schindler B. D., Kaatz G. W. (2016). Multidrug efflux pumps of Gram-positive bacteria. *Drug Resistance Updates*.

[9] Yoshida H., Bogaki M., Nakamura S., Ubukata K., Konno M. (1990). Nucleotide sequence and characterization of the staphylococcus aureus norA gene, which confers resistance to quinolones. *Journal of Bacteriology*.

[10] Kaatz G. W., Seo S. M. (1995). Inducible NorA-mediated multidrug resistance in staphylococcus aureus. *Antimicrobial Agents and Chemotherapy*.

[11] Hsieh P. C., Siegel S. A., Rogers B., Davis D., Lewis K. (1998). Bacteria lacking a multi-drug pump: a sensitive tool for drug discovery. *Proceedings of the National Academy of Sciences of the United States of America*.

[12] Stavri M., Piddock L. J. V., Gibbons S. (2007). Bacterial efflux pump inhibitors from natural sources. *Journal of Antimicrobial Chemotherapy*.

[13] Rana T., Singh S., Kaur N., Pathania K., Farooq U. (2014). A review on efflux pump inhibitors of medically important bacteria from plant sources. *International Journal of Pharmaceutical Sciences Review and Research*.

[14] Diniz-Silva H. T., Magnani M., de Siqueira S., de Souza E. L., de Siqueira-Júnior J. P. (2016). Fruit flavonoids as modulators of norfloxacin resistance in Staphylococcus aureus that overexpress norA. *Journal of Food Science and Technology*.

[15] Menezes-Silva S., Lira N. S., Mangueira do Nascimento Y. (2020). Modulation of drug resistance in Staphylococcus aureus by 132-hydroxy-(132-R/S)-pheophytin isolated from sargassum polyceratium. *Microbial Pathogenesis*.

[16] Seukep A. J., Kuete V., Nahar L., Sarker S. D., Guo M. (2020). Plant-derived secondary metabolites as the main source of efflux pump inhibitors and methods for identification. *Journal of Pharmaceutical Analysis*.

[17] Muniz D. F., dos Santos Barbosa C. R., de Menezes I. R. A. (2021). In vitro and in silico inhibitory effects of synthetic and natural eugenol derivatives against the NorA efflux pump in staphylococcus aureus. *Food Chemistry*.

[18] dos Santos Barbosa C. R., Scherf J. R., de Freitas T. S. (2021). Effect of carvacrol and thymol on NorA efflux pump inhibition in multidrug-resistant (MDR) staphylococcus aureus strains. *Journal of Bioenergetics and Biomembranes*.

[19] da Costa R. H. S., Rocha J. E., de Freitas T. S. (2021). Evaluation of antibacterial activity and reversal of the NorA and MepA efflux pump of estragole against staphylococcus aureus bacteria. *Archives of Microbiology*.

[20] de Araújo A. C. J., Freitas P. R., dos Santos Barbosa C. R. (2021). In vitro and in silico inhibition of staphylococcus aureus efflux pump NorA by *α*-pinene and limonene. *Current Microbiology*.

[21] de Menezes I. A., Coutinho H. M., Pinheiro P. (2021). Antibacterial activity and inhibition against Staphylococcus aureus NorA efflux pump by ferulic acid and its esterified derivatives. *Asian Pacific Journal of Tropical Biomedicine*.

[22] Silva P. T. d., Freitas T. S. d., Sena D. M. (2020). Structural, vibrational and electrochemical analysis and antibacterial potential of isomeric chalcones derived from natural acetophenone. *Applied Sciences*.

[23] Tintino S. R., Souza V. C. A. d., Silva J. M. A. d. (2020). Effect of vitamin K3 Inhibiting the function of NorA efflux pump and its gene expression on staphylococcus aureus. *Membranes*.

[24] Schindler B. D., Jacinto P., Kaatz G. W. (2013). Inhibition of drug efflux pumps in staphylococcus aureus: current status of potentiating existing antibiotics. *Future Microbiology*.

[25] Costa L. M., de Macedo E., Oliveira F. A. A. (2016). Inhibition of the NorA efflux pump of staphylococcus aureus by synthetic riparins. *Journal of Applied Microbiology*.

[26] Faillace M. S., Alves Borges Leal A. L., Araújo de Oliveira Alcântara F. (2021). Inhibition of the NorA efflux pump of S. aureus by (Z)-5-(4-fluorobenzylidene)-Imidazolidines. *Bioorganic & Medicinal Chemistry Letters*.

[27] Abu-Hashem A. A., El-Shazly M. (2015). Synthesis, reactions and biological activities of furochromones: a review. *European Journal of Medicinal Chemistry*.

[28] Abu-Hashem A. A., Youssef M. M. (2011). Synthesis of new visnagen and khellin furochromone pyrimidine derivatives and their anti-inflammatory and analgesic activity. *Molecules*.

[29] Elgazwy A.-S. S. H., Edrees M. M., Ismail N. S. M. (2012). Molecular modeling study bioactive natural product of khellin analogues as a novel potential pharmacophore of EGFR inhibitors. *Journal of Enzyme Inhibition and Medicinal Chemistry*.

[30] Borges M. L., Latterini L., Elisei F. (2008). Photophysical properties and photobiological activity of the furanochromones visnagin and khellin. *Photochemistry and Photobiology*.

[31] Vedaldi D., Caffieri S., Dall’Acqua F., Andreassi L., Bovalini L., Martelli P. (1988). Khellin, a naturally occurring furochromone, used for the photochemotherapy of skin diseases: mechanism of action. *Farmaco Sci*.

[32] Anrep G. V., Barsoum G. S., Kenawy M. R., Misrahy G. (1946). Ammi visnaga in the treatment of the anginal syndrome. *Heart*.

[33] Anrep G. V., Kenawy M. R., Barsoum G. S. (1949). The coronary vasodilator action of khellin. *American Heart Journal*.

[34] Vanachayangkul P., Byer K., Khan S., Butterweck V. (2010). An aqueous extract of Ammi visnaga fruits and its constituents khellin and visnagin prevent cell damage caused by oxalate in renal epithelial cells. *Phytomedicine*.

[35] Sharifi-Rad J., Cruz-Martins N., López-Jornet P. (2021). Natural coumarins: exploring the pharmacological complexity and underlying molecular mechanisms. *Oxidative Medicine and Cellular Longevity*.

[36] Araújo R. S., Barbosa-Filho J. M., Scotti M. T. (2016). Modulation of drug resistance in Staphylococcus aureus with coumarin derivatives. *Scientifica*.

[37] Madeiro S. A. L., Borges N. H. P. B., Souto A. L., de Figueiredo P. T., Siqueira-Junior J. P., Tavares J. F. (2017). Modulation of the antibiotic activity against multidrug resistant strains of coumarins isolated from Rutaceae species. *Microbial Pathogenesis*.

[38] Stermitz F. R., Lorenz P., Tawara J. N., Zenewicz L. A., Lewis K. (2000). Synergy in a medicinal plant: antimicrobial action of berberine potentiated by 5ʹ-methoxyhydnocarpin, a multidrug pump inhibitor. *Proceedings of the National Academy of Sciences of the United States of America*.

[39] Neyfakh A. A., Borsch C. M., Kaatz G. W. (1993). Fluoroquinolone resistance protein NorA of Staphylococcus aureus is a multidrug efflux transporter. *Antimicrobial Agents and Chemotherapy*.

[40] Waterhouse A., Bertoni M., Bienert S. (2018). Swiss-Model: homology modelling of protein structures and complexes. *Nucleic Acids Research*.

[41] Chen V. B., Arendall W. B., Headd J. J. (2010). MolProbity: all-atom structure validation for macromolecular crystallography. *Acta Crystallographica Section D Biological Crystallography*.

[42] Trott O., Olson A. J. (2010). AutoDock Vina: improving the speed and accuracy of docking with a new scoring function, efficient optimization, and multithreading. *Journal of Computational Chemistry*.

[43] Ríos J., Recio M. (2005). Medicinal plants and antimicrobial activity. *Journal of Ethnopharmacology*.

[44] Alves Borges Leal A. L., Teixeira da Silva P., Nunes da Rocha M. (2021). Potentiating activity of norfloxacin by synthetic chalcones against NorA overproducing Staphylococcus aureus. *Microbial Pathogenesis*.

[45] da Silva P., da Cunha Xavier J., Freitas T. S. (2021). Synthesis, spectroscopic characterization and antibacterial evaluation by chalcones derived of acetophenone isolated from croton anisodontus Müll.Arg. *Journal of Molecular Structure*.

[46] Rezende-Júnior L. M., Andrade L. M. d. S., Leal A. L. A. B. (2020). Chalcones isolated from arrabidaea brachypoda flowers as inhibitors of NorA and MepA multidrug efflux pumps of staphylococcus aureus. *Antibiotics*.

[47] Falcão-Silva V. S., Silva D. A., Souza M. D. F. V., Siqueira-Júnior J. P. (2009). Modulation of drug resistance in Staphylococcus aureus by a kaempferol glycoside from herissantia tiubae (malvaceae). *Phytotherapy Research*.

[48] Maia G. L. d. A., Falcão-Silva V. d. S., Aquino P. G. V. (2011). Flavonoids from praxelis clematidea R.M. king and robinson modulate bacterial drug resistance. *Molecules*.

[49] Silva H. C., Leal A. L. A. B., Oliveira M. M. (2020a). Stuctural characterization, antibacterial activity and NorA efflux pump inhibition of flavonoid fisetinidol. *South African Journal of Botany*.

[50] e Silva A. K., dos Reis A. C., Pinheiro E. E. A. (2021). Modulation of the drug resistance by platonia insignis mart. extract, ethyl acetate fraction and morelloflavone/volkensiflavone (biflavonoids) in staphylococcus aureus strains overexpressing efflux pump genes. *Current Drug Metabolism*.

[51] Braga Ribeiro A. M., Sousa J. N. d., Costa L. M. (2019). Antimicrobial activity of phyllanthus amarus schumach. & thonn and inhibition of the NorA efflux pump of staphylococcus aureus by phyllanthin. *Microbial Pathogenesis*.

[52] Kaatz G. W., Seo S. M., Ruble C. A. (1993). Efflux-mediated fluoroquinolone resistance in Staphylococcus aureus. *Antimicrobial Agents and Chemotherapy*.

[53] Samosorn S., Tanwirat B., Muhamad N. (2009). Antibacterial activity of berberine-NorA pump inhibitor hybrids with a methylene ether linking group. *Bioorganic & Medicinal Chemistry*.

[54] Markham P. N., Westhaus E., Klyachko K., Johnson M. E., Neyfakh A. A. (1999). Multiple novel inhibitors of the NorA multidrug transporter of staphylococcus aureus. *Antimicrobial Agents and Chemotherapy*.

[55] Oliveira M. M., Santos H. S., Coutinho H. D. M. (2020). Spectroscopic characterization and efflux pump modulation of a thiophene curcumin derivative. *Journal of Molecular Structure*.

[56] Dantas N., de Aquino T. M., de Araújo-Júnior J. X. (2018). Aminoguanidine hydrazones (AGH’s) as modulators of norfloxacin resistance in Staphylococcus aureus that overexpress NorA efflux pump. *Chemico-Biological Interactions*.

[57] Best M. M. (1952). Management of khellin toxicity; effects of dosage and purification. *The American Journal of the Medical Sciences*.

[58] Risaliti L., Yu X., Vanti G., Bergonzi M. C., Wang M., Bilia A. R. (2021). Hydroxyethyl cellulose hydrogel for skin delivery of khellin loaded in ascosomes: characterization, in vitro/in vivo performance and acute toxicity. *International Journal of Biological Macromolecules*.

[59] Khalil N., Bishr M., El-Degwy M., Abdelhady M., Amin M., Salama O. (2021). Assessment of conventional solvent extraction vs. supercritical fluid extraction of khella (Ammi visnaga L.) furanochromones and their cytotoxicity. *Molecules*.

