# Supplementary material for: Modulation of Drug Resistance by Furanochromones in NorA Overexpressing Staphylococcus Aureus
Source: Evid Based Complement Alternat Med. 2022 Sep 17;2022:9244500. doi: 10.1155/2022/9244500 (PMC9509268; doi:10.1155/2022/9244500)
Supplement: Supplementary Materials — Figure S1: protein-ligand interaction diagram of piperine on the binding site of the NorA model. Figure S2: protein-ligand interaction diagram of verapamil on the binding site of the NorA model. Figure S3: protein-ligand interaction diagram of reserpine on the binding site of the NorA model. [file 9244500.f1.docx]

**Modulation of drug resistance by furanochromones in *norA* overexpressing *Staphylococcus aureus***

Runnig title: Modulation of drug resistance by furanochromones

*Damara F. Rodrigues^1^, Nathalie H. P. B. Borges^1^, Carlos Emídio S. Nogueira^2^, Josean F. Tavares^3^,* *Daniel Dias Rufino Arcanjo^4^, Humberto M. Barreto^5*^, José Pinto de Siqueira-Júnior^1^*

*
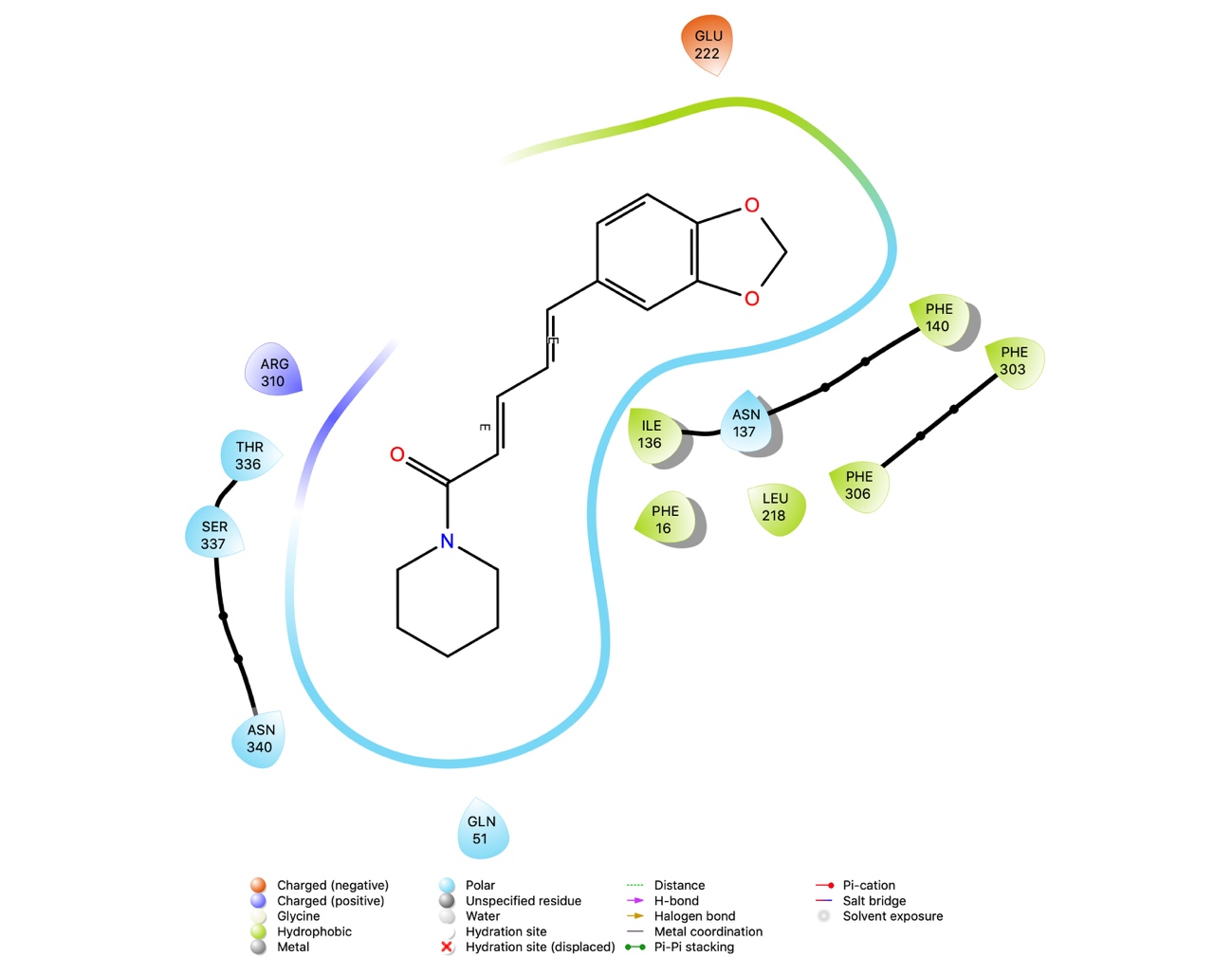
*

Figure S1. Protein-Ligand interaction diagram of Piperine on the binding site of the NorA model.


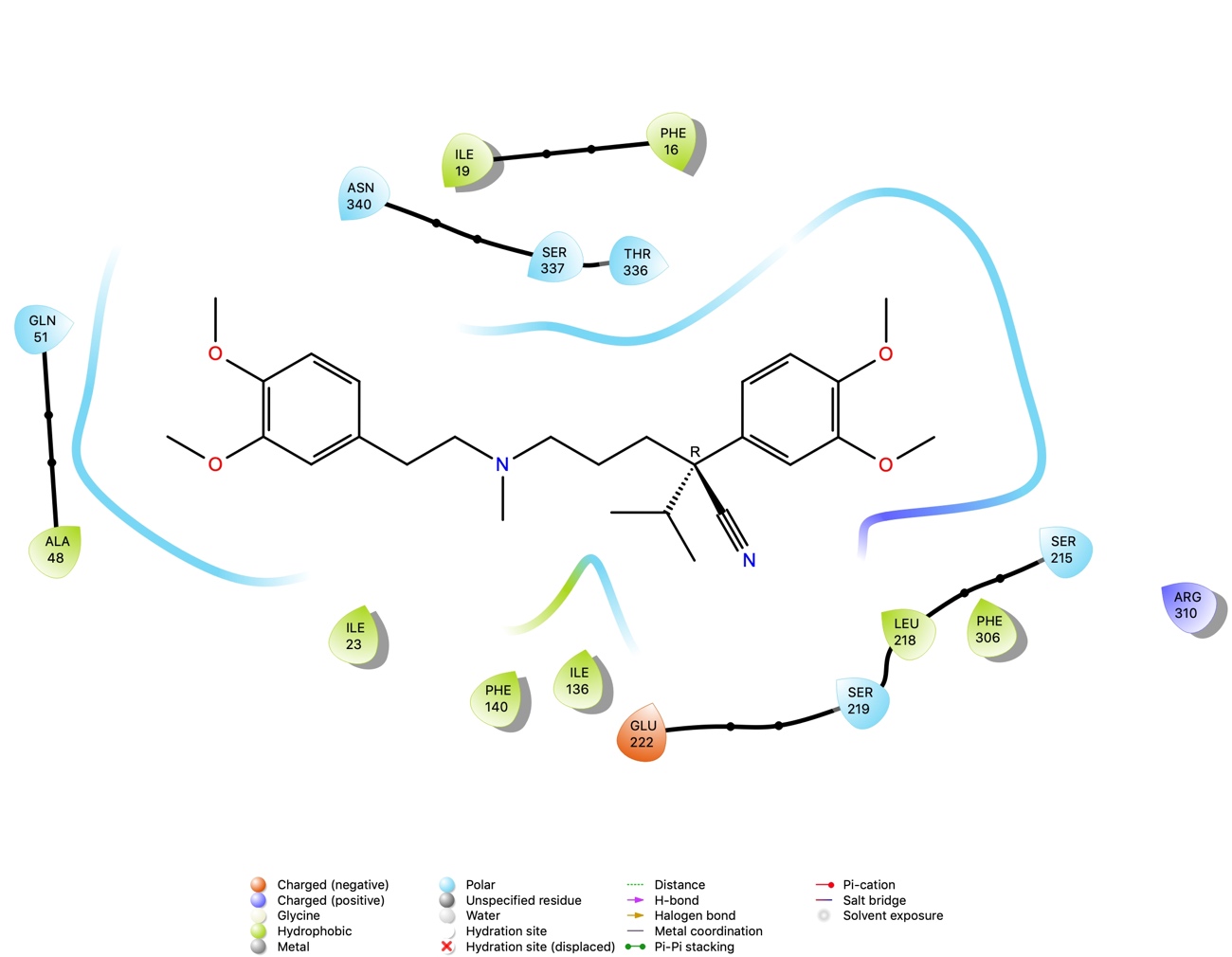


Figure S2. Protein-Ligand interaction diagram of Verapamil on the binding site of the NorA model.


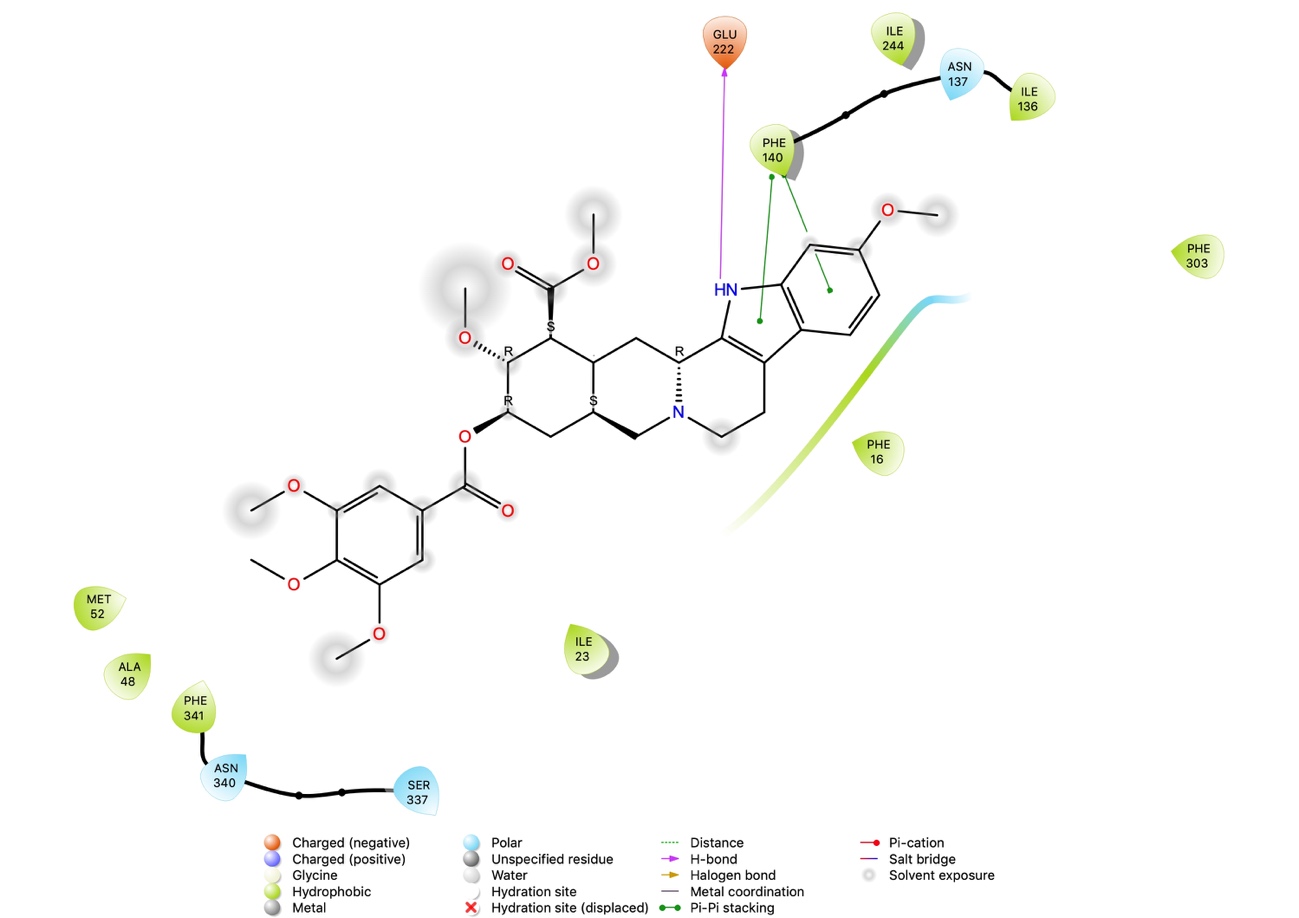


Figure S3. Protein-Ligand interaction diagram of Reserpine on the binding site of the NorA model.
